# Effects of the Ionic and Nanoparticle Forms of Cu and Ag on These Metals’ Bioaccumulation in the Eggs and Fry of Rainbow Trout (*Oncorhynchus mykiss* W.)

**DOI:** 10.3390/ijerph17176392

**Published:** 2020-09-02

**Authors:** Monika Kowalska-Góralska, Magdalena Senze, Joanna Łuczyńska, Katarzyna Czyż

**Affiliations:** 1 Institute of Animal Breeding, Faculty of Biology and Animal Sciences, Wroclaw University of Environmental and Life Sciences, 51-630 Wroclaw, Poland; magdalena.senze@upwr.edu.pl (M.S.); katarzyna.czyz@upwr.edu.pl (K.C.); 2 Faculty of Food Sciences, University of Warmia and Mazury in Olsztyn, 10-726 Olsztyn, Poland; jlucz@uwm.edu.pl

**Keywords:** nanometals, ions, copper, silver, bioaccumulation, rainbow trout, eggs, fry

## Abstract

Nanotechnology is a rapidly growing field of science, and an increasing number of nanoproducts, including nanometals, can be found on the market. Various nanometals and the products that are manufactured based on them can help to fight bacteria and fungi, but they can also penetrate organisms and accumulate in them. This study aimed to compare the effects of two metals, silver (Ag) and copper (Cu), with known antibacterial and fungicidal properties in their ionic (AgNO_3_, CuSO_4_·5H_2_O) and nanoparticle (AgNPs, CuNPs) forms on rainbow trout eggs and fry. Concentrations of metals ranging from 0 to 16 mg/L were used during egg swelling for 2 h. The swelling of eggs in Cu solutions resulted in an increase in Cu content in the eggs (just like in the case of Ag); however, the changes in fry were not significant in the case of both Ag and Cu. The concentrations of these metals in eggs was greatly affected by the applied form of Ag and Cu. Because CuNPs penetrated the embryo in fish eggs at lower concentrations compared to AgNPs, it would be worth considering them for antibacterial applications during egg incubation.

## 1. Introduction

Nanotechnology is a field of science that has been developing rapidly in recent years. The distinguishing feature of nanoelements is that one of their dimensions does not exceed 100 nm, and due to their size and relatively large surface area to volume ratio, their properties differ compared to the ionic forms of the same elements [1,2]. Besides the many benefits in different areas of life and industry, nanotechnology can also present certain risks to the environment [3,4]. This particularly concerns the aquatic environment, which receives significant amounts of pollutants from industrial discharge. On the other hand, nanoproducts can also be beneficial for flowing water fish farmers in the fight against *Saprolegnia* sp., but they may concurrently pose a hazard when nanoelements are accumulated [5].

Farmers of salmonid fish have been struggling for many years with problems related to hatching. The conditions for egg incubation and the long time required for this process affect the development of *Saprolegnia* sp. fungi [6]. Malachite green, a seemingly ideal agent, was used for this purpose for some time; however, when its harmfulness to people, including its carcinogenic effects, was determined, the search for substitutes started. Attempts were made to use formalin, H_2_O_2_, NaCl, or even iodine in the fight against *Saprolegnia* [6]; however, there is still a need to search for alternatives to replace malachite green [7]. Literature data indicate that nanoelements can be harmful to living organisms [5,8,9,10], while on the other hand, their properties, e.g., bacteriological or fungicidal properties, may be very beneficial. Research on antifungal applications of nanoelements has been performed by many authors. Zhang et al. [11] examined the antibacterial effect of nanoparticles on *Aspergillus niger* and *Penicillium citrinum*. Rai et al. [12] and Barrena et al. [13] have discussed the antifungal and antibacterial properties of Ag. On the other hand, Korzeniowski and Wiweger [14] drew attention to the occurrence of *Pseudoloma neurophilia* (microsporidiosis) in zebrafish (*Danio rerio*) breeding and related problems, which additionally indicates the need for effective antibacterial preparations in fish farming.

According to Shahbazzadeh [15], nanosilver (as Nanocid) at low concentrations is not toxic to rainbow trout; therefore, it is worth considering the possibility of its application as a substitute for other agents, e.g., the abovementioned malachite green, chloramine T, or copper sulfate. In turn, in a study on rainbow trout hatchability, Soltani et al. [16] drew attention to the possibility of counteracting saprolegniosis using a preparation with silver nanoparticles (AgNPs), where the authors obtained promising results using doses of 0.5–4 mg/L. Although the highest concentration used during the egg incubation resulted in a worse percentage onset, the outcomes were very beneficial compared to the control group. When using AgNPs concentrations of 1, 2, and 4 mg/L, the percentage of hatchability of rainbow trout eggs was only slightly worse than after the application of malachite green [16]. Besides silver nanoparticles, copper also inhibits the growth of bacteria, such as *Escherichia coli* and *Bacillus subtilis* [17]. Copper in the form of copper sulfate was also used as a disinfectant, and its nano form exhibits similar properties. The sensitivity of fish organisms to toxic substances depends on their age, like in the case of other animals, where the youngest individuals are the most sensitive [18]. Moreover, the study by Shaw and Handy [19] suggests that certain nanometals may penetrate the chorion and be more harmful to fish than dissolved forms.

Therefore, carrying out a study on the effect of applying AgNPs and its most popular ionic form AgNO_3_ to fish eggs is justified. Additionally, due to the similar antibacterial and antifungal properties of copper in the form of CuSO_4_ and CuNPs, these metals should also be examined. Thus, this study aimed to compare the effects of different doses of AgNPs and CuNPs, as well as easily soluble salts (AgNO_3_ and CuSO_4_·5H_2_O) on the elemental concentrations in the eggs and fry of rainbow trout (*Oncorhynchus mykiss* Walb.).

## 2. Materials and Methods

The following Cu and Ag compounds were used to prepare the solutions of CuNPs, CuSO_4_, AgNPs, and AgNO_3_: Cu < 100 nm nanopowder from Sigma Aldrich Ltd. (Poznan, Poland) (<100 nm (BET—Brunauer-Emmett-Teller), <3% oxygen passivation, 99% trace metals basis), CAS number: 7440-50-8; CuSO_4_ (CuSO_4_·5H_2_O, described as CuSO_4_; purity > 98%) from Sigma Aldrich Ltd., CAS number: 7758-99-8; Ag <100 nm from Nanoco Ltd. (Katowice, Poland); AgNO_3_ salt, CAS number: 7761-88-8. The solutions of chemical compounds were prepared by taking into account the metal concentration. The copper solutions were made just before the application.

Prior to dilution, the copper solutions were subjected to a sonication process [8,20] using a SONIC-5 apparatus (POLSONIC, Warsaw, Poland) at 50 Hz and 620 W. This method applies ultrasonic waves of different intensities, where the energy carried by each of the waves allows for breaking intermolecular bonds; activating the chemical reactions, porosity, and homogenization of the material; preventing the agglomeration of the metals present in the nano form. The latter property was used to obtain homogeneous mixtures (solutions). In this way, the composition of the solution in each site was identical regardless of the sample size. The process took 30 min each time.

Rainbow trout eggs (*Oncorhynchus mykiss* Walb.) were obtained from four females and sperm from six males was poured over the eggs. The group was then divided into individual research subgroups. A total of three repetitions of six study groups containing different concentrations of the analyzed metals were created: 0, 1, 2, 4, 8, and 16 mg/L, that is, 18 subgroups for each of the four chemicals (AgNPs, AgNO_3_, CuNPs, and CuSO_4_). A total of 72 subgroups were created. There were 200 eggs in each subgroup.

The egg incubation was carried out in the long-flow devices of the Fish Stocking Center in Szczodre (Poland) in a separate basket for each subgroup. The flow and all treatments were carried out in all the instruments during the egg incubation.

The process of egg swelling in each group was carried out within 2 h at 10 °C in small plastic bowls (polypropylene) in the applied solutions. During this time, the eggs were exposed to AgNPs, AgNO_3_, CuNPs, or CuSO_4_, where the chorion did not provide additional protection for the future embryo [21]; thus, the maximum effect on the egg and its embryo was produced. After 2 h, all eggs were taken out of the AgNP, AgNO_3_, CuNP, and CuSO_4_ solutions and incubated in the same conditions at 11.0 °C, pH 7.5, and 6 mgO_2_/L in water.

After the swelling of the eggs, some of the eggs from each subgroup (12 pieces) were taken to mineralize them and determine their metal content. After hatching, 15 pieces of fry were taken from each subgroup to mineralize them and determine their metal content.

Samples of the eggs and fry (2 g wet weight) were digested with 5 mL 65% HNO_3_ for 24 h at room temperature and were mineralized in a microwave oven (Mars-5, Varian Medical Systems Poland Ltd., Warsaw, Poland). The mineralization was conducted in two stages: the first stage lasted 45 min at 100 °C, while the second stage lasted 10 min at 200 °C.

The cooled samples were transferred into 15 mL Teflon flasks, and the Cu and Ag concentrations were measured using a Varian SpectrAA-220-FS atomic absorption spectrophotometer (Varian Medical Systems Poland Ltd., Warsaw, Poland). The accuracy of the method was evaluated via calibration versus an international standard (DORM-4, DOLT-4, Community Bureau of Reference (BCR), Brussels, Belgium). The calibration curves were prepared using standard solutions (1000 μg/L) with 0.1 M HNO₃ supplied by J.T.Baker^®^ (Deventer, Netherlands). The calibration curves were linear within the range of the metal concentrations (regression coefficients R² ≥ 0.999). The detection limits (LOD) were 0.01 mg/kg for Ag and 0.05 mg/kg for Cu, and the sensitivities were 0.01 mg/L and 0.02 mg/L, respectively.

Statistical analyses were performed using the R package (Version 1.1.442, RStudio, Inc., Boston, MA, USA), where the normality of the distribution was tested using the Shapiro–Wilk test. Due to the uneven normality, a non-parametric analysis was performed using the Kruskal–Wallis and the Wilcoxon post hoc tests, in which all groups were compared. Different metal concentrations were also compared in the egg groups and separately in the fry groups, as well as the type of chemical compound in the egg groups and separately in the fry groups. The significance level was set at 0.05. A permutation test with 1000 permutations was performed. An examination of the differences in the Cu and Ag concentrations of the swelling eggs were performed for all studied groups, regardless of the metal used, i.e., AgNPs, AgNO_3_, CuNPs, and CuSO_4_, taking into account the effect of their concentration or life stages (eggs/fry); these differences were tested via the PERMANOVA test (1000 repetitions) using the Vegan 2.4 package for R [20]. The principal component analysis (PCA) was carried out using the Factoextra package for R [22].

## 3. Results

### 3.1. Influence of Silver Ions and Silver Nanoparticles on the Silver Concentration in the Rainbow Trout Eggs and Fry

The swelling of the eggs in the silver solutions affected the silver content in the rainbow trout eggs and the fry. The silver concentrations in the eggs are presented in Table 1. The greatest differences were found between the Ag concentration and the type of chemical compound used. The application of AgNO_3_ had a greater influence on the changes in the silver content in the eggs compared to AgNPs.

The application of AgNO_3_ increased the Ag content in the group of fry for the concentrations of 4, 8, and 16 mg/L (Table 1). In the case of AgNPs, these values in the fry were higher only for concentrations of 8 and 16 mg/L (Table 1). Regarding the 0 mg/L (control) group, a higher percentage of the changes was observed in the eggs compared to the fry (Table 1).

After the application of AgNO_3_, higher concentrations of Ag were found in the eggs compared to the AgNP application (Table 1), where the differences were statistically significant (Table 2). Significantly lower Ag contents were observed in the fry compared to the eggs, regardless of the type of compound used (Table 2). The Cu content in the fry was similar and the differences were statistically insignificant, regardless of the type of chemical used (Table 2).

Permutations allowed for determining the significance between the Ag concentration in eggs or fry and the chemical compounds (AgNPs, AgNO_3_), concentrations (0, 1, 2, 4, 8, 16 mg/L), and fish life stage (egg or fry). As a result of the analysis, it was demonstrated that all the examined factors had a significant (*p* < 0.001) effect on the Ag concentration in the subjects studied, and the relationships between these factors were equally important (Table 3).

**Figure 1:**
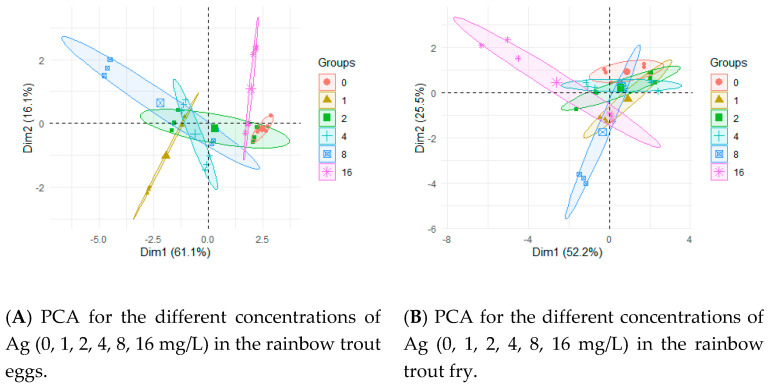

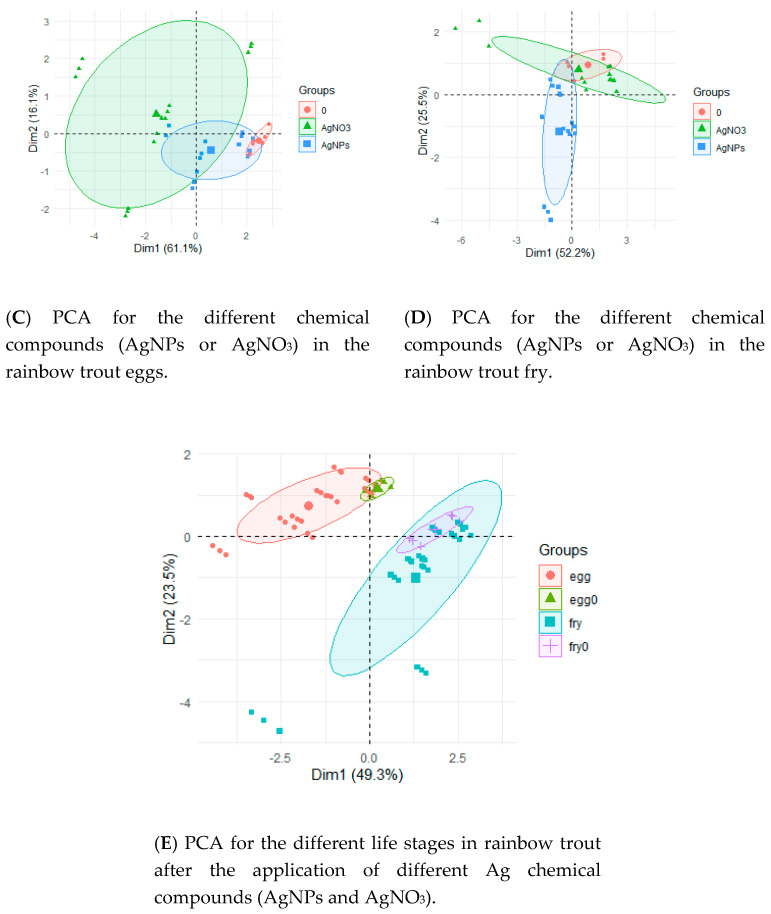
Principal component analysis (PCA) by groups according to life stage (eggs, fry) of the rainbow trout, chemical compound (AgNPs, AgNO_3_), and Ag concentration (0, 1, 2, 4, 8, 16 mg/L). Dim—dimension.

The results of the PCA are presented in Figure 1. The applied concentrations of AgNPs or AgNO_3_ (Figure 1A,B) affected the Ag concentration in the eggs and fry to a lower degree than the applied form of silver (Figure 1C,D). The differences were clearly visible during the egg testing and became less pronounced in the case of fry. The differences between the Ag concentrations in different life stages (eggs, fry) are clearly visible (Figure 1E).

**Figure 2:**
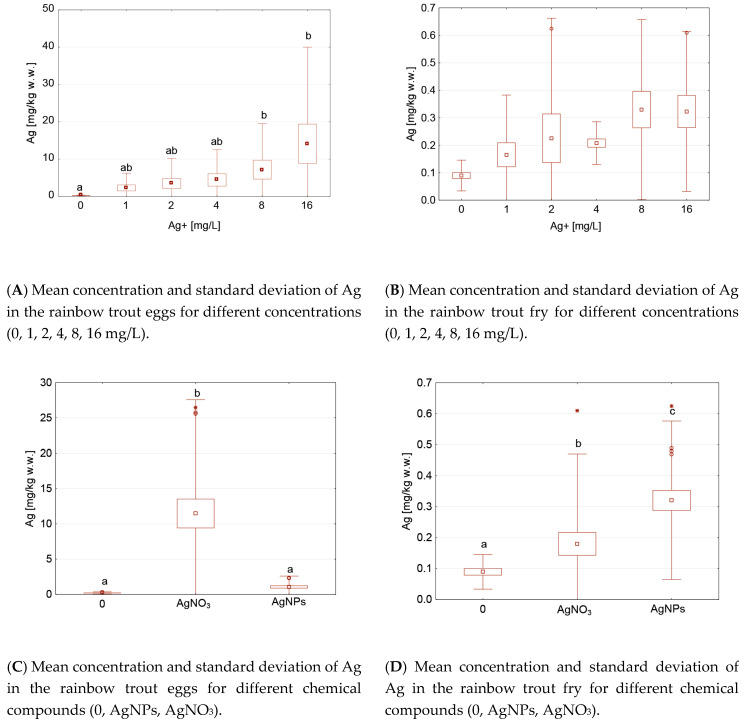
Mean and standard deviation of Ag concentrations in the rainbow trout groups after the Ag applications, depending on the life stage (eggs or fry), chemical compound (0 (control), AgNPs, AgNO_3_), and Ag concentration (0, 1, 2, 4, 8, 16 mg/L). Different letters indicate statistical differences (*p* < 0.05) between groups.

Statistically significant differences were found in the egg groups between the control group and Ag concentrations of 8 or 16 mg/L, regardless of the form of the silver, i.e., AgNPs or AgNO_3_ (Figure 2). These differences were not demonstrated for the Ag concentration in the fry (Figure 2B). Statistical differences in the Ag concentration in the eggs (Figure 2C) and fry (Figure 2D) were found between the control group and after the AgNO_3_ application (Figure 2). On the other hand, the differences between the Ag concentration in the eggs in the control group and the groups after applying the AgNPs were not significant in the eggs (Figure 2C) and fry (Figure 2D). A statistically significant positive correlation was found between the Ag content in the eggs and fry and the concentration (0, 1, 2, 4, 8, 16 mg/L) of Ag, and between the concentration of Ag (0, 1, 2, 4, 8, 16 mg/L) and the applied chemical (AgNPs or AgNO_3_) (Table 4).

### 3.2. Influence of Copper Ions and Copper Nanoparticles on the Copper Concentration in Rainbow Trout Eggs and Fry

The swelling of the eggs in the copper solutions resulted in increased copper content in the eggs. This was particularly evident after the application of CuSO_4_. These differences were directly proportional to the applied CuSO_4_ concentration (Table 5). The differences in the Cu concentrations in the eggs after the CuNP application were most noticeable in the case of the 8 and 16 mg/L concentrations (Table 5). These changes were not observed in the fry after hatching (Table 5), regardless of the chemical used. Relative to the control group, a higher change percentage was observed in the eggs than in the fry, and a higher change percentage was observed due to the CuSO_4_ than the CuNPs (Table 5).

Although higher Cu concentrations were observed in the eggs (Table 5), the type of compound used was of no significance here (Table 6). The concentrations of Cu in the fry after the application of different types of compounds were lower compared to the eggs and independent of the compound used (Table 6).

A statistically significant positive correlation was found between the Cu content in the eggs or fry and the Cu concentration during swelling (0, 1, 2, 4, 8, 16 mg/L), and a statistically significant negative correlation was found between the Cu concentration in the eggs or fry and the life stages (eggs, fry) (Table 7), whereas no statistically significant correlations were found between the other factors.

Permutations allowed for determining the effect of the significance between the Cu concentration in the eggs or fry and the chemical compounds (CuNPs, CuSO_4_), concentrations (0, 1, 2, 4, 8, 16 mg/L), and life stage (egg or fry). As a result of the analysis, it was found that all the examined factors had a significant (*p* < 0.001) effect on the Cu concentration in the subjects studied, and the relationships between these factors were equally important (Table 8).

**Figure 3:**
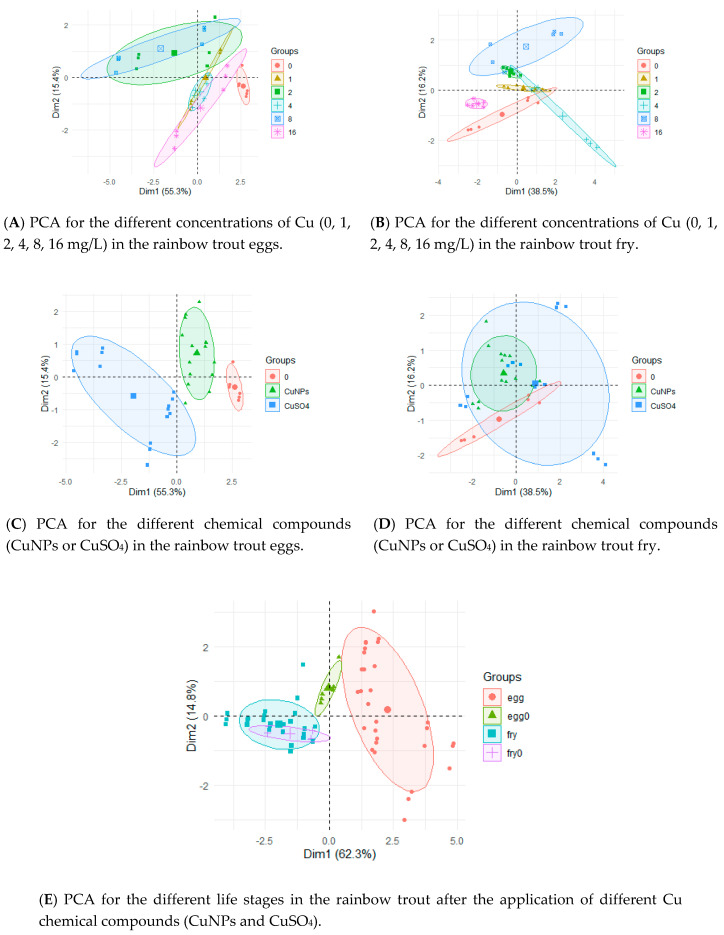
PCA by groups according to the life stage (eggs, fry) of the rainbow trout, chemical compound (CuNPs, CuSO_4_), and Cu concentration (0, 1, 2, 4, 8, 16 mg/L).

The PCA results are presented in Figure 3 The concentrations had a lower effect on the eggs and fry (Figure 3A,B) than the applied copper form (Figure 3C,D). Similar to the case of silver, the differences between the metal concentrations in the eggs and fry (Figure 3E) are clearly visible in the case of copper.

**Figure 4:**
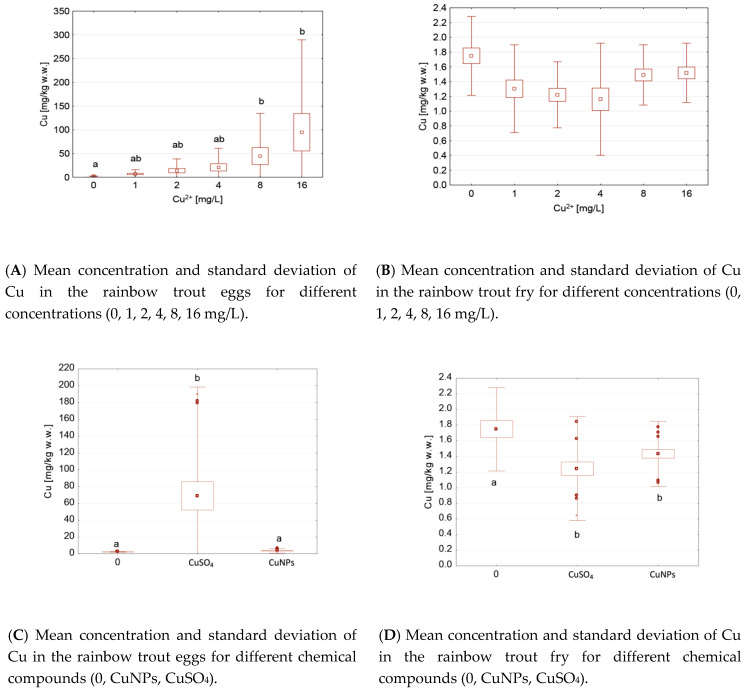
Mean and standard deviation of the Cu concentrations in the rainbow trout groups after the Cu application, depending on the life stage (eggs or fry), chemical compound (0 (control), CuNPs, CuSO_4_), and Cu concentration (0, 1, 2, 4, 8, 16 mg/L). Different letters indicate statistical differences (*p* < 0.05) between groups.

Statistically significant differences were found in the eggs between the control groups and the groups with 8 and 16 mg/L of Cu (Figure 4A), and also between the control groups and after the CuSO_4_ application (Figure 4C). There was a statistically lower Cu concentration in the fry (Figure 4D) and no differences in the Cu concentration (0, 1, 2, 4, 8, 16 mg/L) in each of the fry groups. In contrast, the differences between the control and CuNPs were not significant in the eggs but were significant in the fry (Figure 4C,D). The differences between the CuNPs and CuSO_4_ were noted in the eggs (Figure 4C) but not in the fry (Figure 4D). The differences between the Cu concentration in the different life stages (eggs, fry) are clearly visible (Figure 3E).

**Figure 5:**
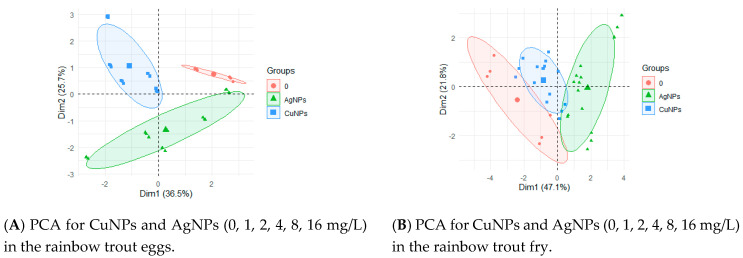
PCA by groups, depending on the fish life stage and the type of nanometal used.

Taking into account the results of PCA and considering only the nanoelements, i.e., AgNPs and CuNPs, attention should be paid to the different effects of these compounds on the eggs (Figure 5A) and fry (Figure 5B).

**Figure 6:**
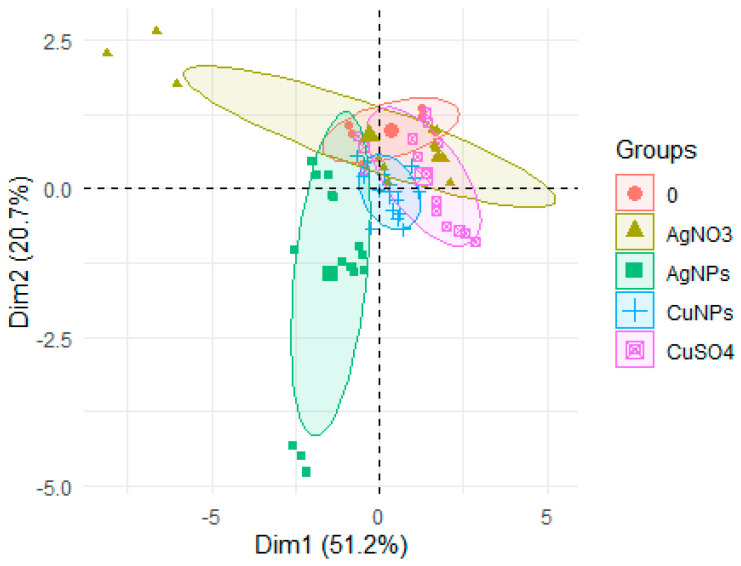
PCA for different chemical compounds (AgNO_3_, AgNPs, CuSO_4_, CuNPs) in the rainbow trout eggs and fry.

Taking only hatching into account to determine the effect of the applied preparation (CuNPs, CuSO_4_) on the changes in its metal content, it can be observed with more than a 70% probability that the NP forms had a lower influence on hatching than the applied Ag forms (Figure 6).

## 4. Discussion

Choi et al. [23,24] and Jayesh et al. [25] drew attention to the antibacterial properties of silver, which was known for many years. Although no physiological significance is attributed to silver, it can be accumulated in organisms after its application. The toxicity of silver to rainbow trout was demonstrated by Al.-Bairuti et al. [26], who observed an increase in silver content in the eggs, depending on silver concentration; similar tendencies were noted by Morgan and Wood [27]. In our study, the levels of silver accumulation were also dependent on the type of chemical compound used. Higher contents of Ag in the eggs were found after an application of AgNO_3_ compared to AgNPs. This is not consistent with the results of Farkas et al. [28], who indicated that Ag ions (e.g., AgNO_3_) have a lower effect on the integrity of the membrane. It is possible that the application of Ag during egg swelling limited or even stopped the metal penetration through the chorion into the egg. However, it is likely that the amount of silver that penetrated the embryo was low, as there were no statistically significant differences in the concentration of silver in the fry. However, this may also indicate that most of the silver stayed in the chorion or the liquid and not in the embryo. The application of AgNO_3_ affected the silver accumulation to a greater extent than in the case of AgNPs. Differences between the interactions of AgNO_3_ and AgNPs were pointed out by Farkas et al. [28] in their study on rainbow trout hepatocytes regarding reactive oxygen species (ROS), and Gange et al. [29], who pointed out the higher bioavailability of Ag ions, which may explain its higher concentration in the eggs of rainbow trout compared to the analogous concentrations of AgNPs, which was also noted in our study. As in our study, Griffitt et al. [30] drew attention to a significantly different LC_50_ value for zebra fish (*Danio rerio*) in the case of AgNP and Ag+ applications, where the ionic forms were more harmful; this was also confirmed by Shaw et al. [31] and Salari et al. [32]. In our study, the silver content in the eggs or fry was also the highest after the AgNO_3_ application.

Compared to the control group, the percentage change in the CuNP content was lower than that of the AgNPs in the eggs, just like in the fry. This is not consistent with the results of the study conducted by Ostaszewska et al. [33], who demonstrated that CuNPs were more toxic than AgNPs. This was confirmed by the results of the study by Shahbazzadeh et al. [15], who investigated the effect of AgNPs on rainbow trout. However, the difference between these studies and ours is the different methods used for the metal application, which could have produced the difference in results. The higher reactivity associated with the surface area of NPs was noted by Pulit-Prociak et al. [34]. Salari et al. [35] demonstrated significant differences in the influence of AgNPs on rainbow trout fry in freshwater compared to water with higher salinity, indicating the aquatic environment affects the toxicity. For this reason, all compounds were simultaneously tested under the same conditions, preventing the influence of different environmental conditions, which also made the interpretation of the results less complicated. Large differences in the metal concentration between the eggs and fry were noted. However, the percentage changes of the metal content were lower after the application of CuNPs compared to AgNPs.

The antibacterial activity of CuSO_4_ was already demonstrated in previous studies [36,37,38,39]. However, the CuSO_4_ harmfulness was pointed out by Clearwater et al. [40], who stated that it can be bioaccumulated in organisms, which was also noted previously by Kowalska-Góralska [21]. The swelling of the egg in copper solutions increased the copper content in the egg; however, similar to the case of Ag in fry, Cu concentration in fry was not significant; perhaps the absorption pathways through endocytosis described by Isani et al. [38] are hindered for NPs. The amount of metal that permeates from CuNP and AgNP solutions to the embryo seems to be low; therefore, the differences between individual concentrations are not significant in the case of fry. In our study, we applied metals during egg swelling, when the chorion was not a sufficient barrier. We facilitated penetration to check whether the metals could penetrate the hatch. Therefore, our results may differ from the results obtained by Shaw and Handy [19]. However, the study by Shaw and Handy [19] suggests that NPs penetrate through the chorion and the authors also suggest that the NPs display greater harmfulness compared to ionic forms, which was not confirmed by the results of our experiment. In contrast, similar results to ours were obtained by Shaw et al. [31] in a study that demonstrated the higher toxicity of CuSO_4_ than CuNPs for young rainbow trout. According to Black et al. [41], Kong et al. [42], and Ostaszewska et al. [33], Cu is very toxic to fish, which was also confirmed in our study by the higher concentrations of copper in the eggs compared to silver, though the only difference was that it involved the ionic forms of metals in our case. In the case of nanoelements, these differences were much smaller. The low Cu content in the fry after hatching in the solutions of CuNPs and CuSO_4_ indicated its low level of penetration into the fry.

Wang et al. [43] showed that the amount of Cu that accumulates is proportional to the concentration, which was also confirmed in our experiments, but only for the copper concentration in the eggs. Wang et al. [43,44] and Shaw et al. [31] demonstrated that CuSO_4_ is more harmful than CuNPs to eggs, which is consistent with our results.

The form of copper used had a greater effect on the concentration of this metal in the eggs than the other parameters studied (chemical compound, concentration). Similar observations were made by Al-Bairuty et al. [45], who determined a higher accumulation in the case of CuSO_4_ than in the case of CuNPs. Hoseini et al. [20] found a greater effect of CuNPs than the ionic Cu form on plasma alkaline phosphatase (ALP) activity. Admittedly, Bai et al. [46] pointed out that CuNPs can delay embryo hatching if a concentration of >0.1 mg/L is used. The lower concentrations applied in our study did not show increased egg mortality in general; this was only noted in the case of the highest concentration of 16 mg/L.

Various biocidal preparations are constantly being tested [16,47,48,49,50]. Taking into account different studies on egg bathing in malachite green [7] and different forms of AgNPs, and comparing similar concentrations of Ag and Cu in the ionic form and as CuNPs, it can be concluded that the concentrations used by previous researchers significantly influenced the Ag and Cu content in the eggs. The applied concentrations had a lower effect on the eggs in the case of a CuNP application, although CuSO_4_ is a form known for its bactericidal properties since it has a greater effect on the changes in Cu content in eggs. Therefore, in our opinion, it is reasonable to test CuNP and AgNP solutions as antibacterial egg baths. Our results indicate that the Ag forms caused more changes in hatching compared to the control group than the Cu forms. Moreover, a higher percentage of changes was observed in the eggs compared to the fry, regardless of the compound used. In the case of the Ag content in the eggs, its higher level was observed after an application of AgNO_3_ compared with AgNP applications.

## 5. Conclusions

1. The ionic forms of Cu and Ag penetrated the eggs to a higher degree compared to their nano form, which may negatively affect further fish development.
2. The swelling of the eggs in solutions of copper and silver increased the content of the studied metals in the eggs.
3. Due to the concentration of the metals used during hatching, it should be noted that Cu reduced its concentration during hatching compared to the control group, which was not observed for the Ag; this was probably due to Ag more effectively penetrating the embryo inside the eggs.
4. All the studied factors (metal form (ionic and nano), concentration (1, 2, 4, 8, 16 mg/L), and life stage (eggs, fry)) had a significant impact on the metal concentration in the rainbow trout eggs.
5. Further studies on the use of CuNPs as an antibacterial agent during the incubation of rainbow trout eggs are recommended.

## Figures and Tables

**Table 1:** Silver nanoparticles (AgNPs) and ionic Ag (Ag^+^) contents in rainbow trout eggs and fry (mg/kg w.w.) after an application of different concentrations of Ag in the form of AgNO_3_ or AgNPs (mgAg/L).

Chemical Compound	Concentration (mgAg/L)	*N*	Eggs		Fry	
			Ag (Min–Max Average ± SD)	Percentage Relative to the Control	Ag (Min–Max Average ± SD)	Percentage Relative to the Control
-	0	6	0.15–0.36 0.26 ± 0.08	100	0.06–0.14 0.09 ± 0.03	100
AgNO_3_	1	3	3.9–4.3 4.1 ± 0.17	1577	0.04–0.08 0.07 ± 0.02	78
	2	3	6.2–6.8 6.5 ± 0.31	2500	0.03–0.09 0.07 ± 0.03	78
	4	3	7.8–8.4 8.1 ± 0.30	3115	0.17–0.19 0.18 ± 0.01	200
	8	3	12.4–13.4 12.8 ± 0.51	4923	0.17–0.20 0.18 ± 0.02	200
	16	3	25.6–26.5 25.9 ± 0.46	9962	0.28–0.61 0.41 ± 0.18	456
AgNPs	1	3	0.35–0.47 0.42 ± 0.06	162	0.25–0.29 0.26 ± 0.02	289
	2	3	0.35–0.49 0.40 ± 0.07	154	0.26–0.62 0.38 ± 0.21	422
	4	3	0.66–0.72 0.69 ± 0.03	265	0.20–0.26 0.24 ± 0.03	267
	8	3	1.5–1.6 1.5 ± 0.06	577	0.47–0.49 0.48 ± 0.01	533
	16	3	2.3–2.4 2.32 ± 0.03	892	0.23–0.27 0.24 ± 0.02	267

**Table 2:** Significant differences were observed in the Ag contents in rainbow trout eggs and fry after swelling with different concentrations of AgNO_3_ and AgNPs. The statistically significant correlations are marked in bold (*p* < 0.05).

Groups	Z	*p*
Eggs with AgNO_3_ and eggs with AgNPs	**2.02**	**0.043**
Eggs with AgNO_3_ and fry with AgNO_3_	**2.02**	**0.043**
Eggs with AgNO_3_ and fry with AgNPs	**2.02**	**0.043**
Eggs with AgNPs and fry with AgNO_3_	**2.02**	**0.043**
Eggs with AgNPs and fry with AgNPs	**2.02**	**0.043**
Fry with AgNO_3_ and fry with AgNPs	1.48	0.138

**Table 3:** PERMANOVA analysis of the factors (chemical compound, concentration, life stage, and their combinations) affecting the Ag concentration in the fry and eggs of rainbow trout.

Factor	df	MS	F	*p*
Chemical compound	2	2.1380	160.72	0.000999
Concentration	4	0.1333	10.02	0.000999
Life stage (fry/eggs)	1	4.4344	333.35	0.000999
Chemical compound × concentration	4	0.3159	23.75	0.000999
Chemical compound × life stage (fry/eggs)	2	1.3370	100.50	0.000999
Concentration × life stage	4	0.2195	16.50	0.000999
Chemical compound × concentration × life stage (fry/eggs)	4	0.2975	22.36	0.000999

**Table 4:** Spearman correlation results. The statistically significant correlations are marked in bold (*p* < 0.05).

Factor	Ag Concentration
Cu concentration	**0.651907**
Concentration used	**0.484445**
Life stage (eggs/fry)	**0.689679**
Chemical compound	**0.317095**

**Table 5:** Copper nanoparticles (CuNPs) and ions (Cu^+2^) content of the rainbow trout eggs and fry (mg/kg w.w.) after an application of different concentrations of Cu in the forms CuSO_4_ or CuNPs (mgCu/L).

Chemical Compound	Concentration (mgAg/L)	*N*	Eggs		Fry	
			Cu (Min–Max Average ± SD)	Percentage Relative to the Control	Cu (Min–Max Average ± SD)	Percentage Relative to the Control
-	0	6	2.01–2.65 2.44 ± 0.22	100	1.38–2.08 1.75 ± 0.27	100
CuSO_4_	1	3	11.13–11.38 11.25 ± 0.13	461	0.90–1.37 1.18 ± 0.25	67
	2	3	24.98–25.82 25.33 ± 0.44	1038	0.87–1.43 1.16 ± 0.28	66
	4	3	38.82–39.89 39.31 ± 0.54	1611	0.64–1.02 0.85 ± 0.19	49
	8	3	85.00–87.05 85.80 ± 1.10	3516	1.17–1.59 1.42 ± 0.22	81
	16	3	179.1–190.1 183.6 ± 5.72	7525	1.40–1.84 1.62 ± 0.22	93
CuNPs	1	3	2.97–3.23 3.06 ± 0.15	125	1.06–1.70 1.43 ± 0.33	82
	2	3	2.51–2.91 2.69 ± 0.20	110	1.10–1.46 1.28 ± 0.18	73
	4	3	2.56–2.76 2.64 ± 0.11	108	1.28–1.65 1.47 ± 0.19	84
	8	3	3.54–3.79 3.65 ± 0.13	150	1.37–1.78 1.57 ± 0.20	90
	16	3	5.93–6.12 6.00 ± 0.11	246	1.28–1.58 1.42 ± 0.15	81

**Table 6:** Significant differences in the Cu contents in the rainbow trout eggs and fry after swelling with different concentrations of CuSO_4_ and CuNPs. The statistically significant correlations are marked in bold (*p* < 0.05).

Groups	Z	*p*
Eggs with CuSO_4_ and eggs with CuNPs	1.75	0.080
Eggs with CuSO_4_ and fry with CuSO_4_	**2.02**	**0.043**
Eggs with CuSO_4_ and fry with CuNPs	1.75	0.080
Eggs with CuNPs and fry with CuSO_4_	**2.02**	**0.043**
Eggs with CuNPs and fry with CuNPs	**2.02**	**0.043**
Fry with CuSO_4_ and fry with CuNPs	1.21	0.225

**Table 7:** Spearman correlation results. The statistically significant correlations are marked in bold (*p* < 0.05).

Factor	Cu Concentration
Ag concentration	**0.363821**
Concentration used	0.142438
Life stage (eggs/fry)	**−0.864772**
Chemical compound	0.103955

**Table 8:** PERMANOVA analysis of factors (chemical compound, concentration, life stage, and their combinations) affecting the Cu concentration.

Factor	df	MS	F	*p*
Chemical compound	2	1.2989	302.13	0.000999
Concentration	4	0.1298	30.19	0.000999
Life stage (eggs/fry)	1	4.1479	964.81	0.000999
Chemical compound × concentration	4	0.1750	40.70	0.000999
Chemical compound × life stage (eggs/fry)	2	1.1559	268.86	0.000999
Concentration × life stage (eggs/fry)	4	0.1408	32.76	0.000999
Chemical compound × concentration × life stage (eggs/fry)	4	0.1939	45.11	0.000999
